# Transcriptional and Metabolic Changes Following Repeated Fasting and Refeeding of Adipose Stem Cells Highlight Adipose Tissue Resilience

**DOI:** 10.3390/nu16244310

**Published:** 2024-12-13

**Authors:** Natalia M. Galigniana, Mohamed Abdelhalim, Philippe Collas, Thomas Sæther

**Affiliations:** 1 Department of Molecular Medicine, Institute of Basic Medical Sciences, Faculty of Medicine, University of Oslo, 0372 Oslo, Norway; n.m.galigniana@medisin.uio.no (N.M.G.); m.i.m.abdelhalim@medisin.uio.no (M.A.); philippe.collas@medisin.uio.no (P.C.); 2 Department of Immunology and Transfusion Medicine, Oslo University Hospital, 0372 Oslo, Norway

**Keywords:** intermittent fasting, metabolic memory, adipogenesis, resilience

## Abstract

Background: Obesity and related metabolic disorders have reached epidemic levels, calling for diverse therapeutic strategies. Altering nutrient intake, timing and quantity by intermittent fasting seems to elicit beneficial health effects by modulating endocrine and cell signaling networks. This study explores the impact of cyclic nutrient availability in the form of every-other-day fasting (EODF) on human adipose stem cells (ASCs). Methods: We subjected ASCs to repeated fasting/refeeding (F/R) cycles, mimicking low glucose/high fatty acid (LGHF) conditions, and assessed phenotypic and transcriptomic changes, lipid storage capacity, insulin sensitivity, and differentiation potential. Results: Four consecutive F/R cycles induced significant changes in adipogenic gene expression, with upregulation of *FABP4* and *PLIN1* during fasting, and increased lipid storage in the ASCs. Upon differentiation, ASCs exposed to LGHF conditions retained a transient increase in lipid droplet size and altered fatty acid metabolism gene expression until day 9. However, these changes dissipated by day 15 of differentiation, suggesting a limited duration of fasting-induced transcriptional and adipogenic memory. Despite initial effects, ASCs showed resilience, returning to a physiological trajectory during differentiation, with respect to gene expression and lipid metabolism. Conclusions: These findings suggest that the long-term effects of EODF on the ASC niche may be transient, emphasizing the ability of the adipose tissue to adapt and restore homeostasis.

## 1. Introduction

Obesity and associated metabolic disorders have reached epidemic proportions [1]. Obesity has a mixed etiology, and so there is a need for multiple therapeutic strategies and targets. One approach involves intermittent fasting. Changing the quantity and timing of nutrient intake results in beneficial health effects [2,3,4]. The three most common types of intermittent fasting—alternate day fasting (ADF), the 5:2 diet, and time-restricted eating—all result in mild to moderate weight loss (3–8%), over short periods of time (8–12 weeks) in humans [5], while some clinical studies also show reduced blood pressure, LDL-cholesterol, serum triglycerides, and insulin resistance [6,7,8]. Mechanistically, these effects are caused by modulation of endocrine networks, involving insulin and insulin-like growth factor (IGF-1), impinging on cell signaling pathways, and transcriptional rewiring governed by nutrient sensors, such as target of rapamycin (mTOR), AMP-activated protein kinase (AMPK), and sirtuins [3,4,9]. A weight loss regimen in the intermittent fasting ADF category, less studied mechanistically, is every-other-day fasting (EODF), i.e., fasting for 24 h once or twice a week [10,11,12]. How recurrent fasting like EODF affects the differentiation and lipid-storing capacity of in vitro adipose stem cell (ASC) models of adiposity and obesity [13,14,15] is unknown.

Adipose tissue is a dynamic organ with metabolic and endocrine functions [16,17]. In humans, white adipose tissue (WAT) is the main site for storing energy as triacylglycerol (TAG), balancing fluctuations in food availability [18], as well as secreting endocrine factors (adipokines) and metabolites [19,20,21]. Adipose tissue expansion leads to an increased capacity of TAG storage and can take place through an increase in adipocyte size (hypertrophy) and/or through an increase in the number of adipocytes in the tissue (hyperplasia). Each mechanism has a profoundly different effect on metabolic health. The latter form of adaptation relies heavily on adipose stem cells of mesenchymal origin [22,23,24,25] differentiating into mature adipocytes, and preserves healthy tissue functions [26]. On the other hand, adipocyte hypertrophy and ectopic accumulation of TAGs results in impaired adipose tissue function, e.g., altered adipokine secretion [27,28] and low-grade tissue inflammation [29], and consequently promotes metabolic diseases such as insulin resistance [27,29,30]. Therefore, it is important to identify the cues that promote hyperplasia over hypertrophy and vice versa.

One important function of healthy adipose tissue is the ability to respond to fasting by increasing lipolysis, mobilizing fatty acids (FAs) for hepatic β-oxidation [31,32]. Hydrolyzation of TAGs to FAs and glycerol is controlled by phosphorylation of, and interaction between, Hormone Sensitive Lipase (HSL), Perilipin 1 and 5 (PLIN1 and PLIN5), Adipose Triglyceride Lipase (ATGL), and α-β Hydrolase Domain containing 5 (ABHD5) [33,34,35,36]. During lipolysis, the systemic FA concentration can easily reach 500 μM [37,38], and so the local concentration in WAT might be even higher. Fatty acids, especially unsaturated FAs and hydroxyl-FAs [39,40,41], function as endogenous agonists of Peroxisome Proliferator-Activated Receptor gamma (PPARγ). This raises the possibility that high local FA concentrations during fasting might affect FA esterification, storage capacity, and differentiation of ASCs through gene regulation. Whether such changes can permanently affect adipose stem cell fate is unknown.

Here, we investigate the phenotypic and transcriptomic plasticity of ASCs under intermittent nutrient availability and its impact on ASCs’ engagement into adipogenesis. Our results point to a view of ASC resilience, where increased lipid storage and altered gene transcription following intermittent nutrient supply are lost during differentiation, raising the possibility that the long-term effects of EODF might potentially be limited.

## 2. Materials and Methods

### 2.1. Adipose Stem Cells and Differentiation

ASCs were isolated from subcutaneous adipose tissue obtained by liposuction from three healthy women (age 35–45 years, BMI 21–24 kg/m^2^) after informed consent, as previously described [42] and in agreement with the guidelines and regulations of the Regional Committee for Research Ethics for Southern Norway, REK 2013/2102. ASCs were cultured in growth medium consisting of DMEM/F12 (17.5 mM glucose; Cat. 31331, Thermo Fisher Scientific, Waltham, MA, USA) supplemented with 10% fetal bovine serum (FBS; Cat. 10437-028, Thermo Fisher Scientific) and 20 ng/mL basic fibroblast growth factor (FGF2; Cat. PHG0023, Thermo Fisher Scientific). Upon confluency, cells were either subjected to fasting/refeeding cycles or kept in growth medium without FGF2 until such cycles were concluded. Cycled cells were exposed to fasting medium for 24 h, followed by 24 h on refeeding medium. Low-glucose fasting medium consisted of 5 mM glucose DMEM obtained by mixing equal parts of no-glucose DMEM (Cat. 11966, Thermo Fisher Scientific) with F-12 Ham (Cat. 11765, Thermo Fisher Scientific) and adding 2% FBS. In low glucose/high fatty acid fasting medium, 100 or 200 μM oleic acid (Cat. 29557, Cayman Chemical, Ann Arbor, MI, USA) was added. For adipose differentiation, ASCs were induced with a cocktail of 0.5 µM 1-methyl-3 isobutyl xanthine (IBMX; Cat. I5879, Sigma-Aldrich, St. Louis, MO, USA), 1 µM dexamethasone (Cat. D4902, Sigma-Aldrich), 10 µg/mL insulin (Cat. I9278, Sigma-Aldrich) and 200 µM indomethacin (Cat. I7378-56, Sigma-Aldrich) in DMEM/F12 (17.5 mM glucose) with 10% FBS for 9 days, and then continued with only 10 µg/mL insulin for six more days (day 15).

### 2.2. RNA Isolation and qPCR Analysis

Cells were lysed in QIAGEN RLT lysis buffer, and samples stored at −80 °C until further processing. Total RNA was purified using the RNeasy Micro kit (Cat. 74004, Qiagen, Hilden, Germany) and quantified in a Nanodrop ND-1000 spectrophotometer (Saveen Werner, Limhamn, Sweden). Isolated RNA (1 μg) was reverse transcribed using High-Capacity cDNA Reverse Transcription Kit (Cat. 4368814, Thermo Fisher Scientific) with the following conditions: 25 °C for 10 min, 37 °C for 2 h, and 85 °C for 5 min. Real-time qPCR was performed with 1/5 dilution of the resulting cDNA, using IQ SYBR Green Supermix (Cat. 1708882, Bio-Rad Laboratories, Hercules, CA, USA) with the following conditions: 1 × 95 °C for 3 min, before 40 × (95 °C for 10 s and 60 °C for 30 s). Relative fold expression of target genes was calculated as 2^−ΔΔCT^, normalizing to the *SF3A1* gene. All primer pairs showed an efficiency of 90–110% at R^2^ > 0.98. The primers sequences are listed in Supplementary Table S1.

### 2.3. Protein Expression

Cells were washed twice in cold PBS and harvested using lysis buffer (50 mM TrisHCl pH 7.4; 0.27 M sucrose; 1 mM sodium orthovanadate pH 10; 1 mM EDTA; 1 mM EGTA; 10 mM sodium β-glycerophosphate; 50 mM NaF; 5 mM Na_4_P_2_O_7_; 1% Triton X-100; 0.1% β-mercaptoethanol) to analyze protein samples by immunoblotting. Bradford assay (Cat. 5000002, Bio-Rad Laboratories) was used to determine protein concentrations, and 50 ug was loaded to each lane of a pre-cast polyacrylamide gel (Criterion TGX; Cat. 5671034, Bio-Rad Laboratories) to separate proteins using SDS-PAGE. Resolved proteins were transferred onto an Immobilon-FL PVDF membrane (Cat. IPFL00010, Millipore, Burlington, MA, USA), subsequently blocked in 5% BSA for 1 h, and incubated overnight at 4 °C with the following antibodies diluted in PBS with 5% BSA: γ-Tubulin (Cat. T5326, Sigma-Aldrich), phospho-Akt Ser473 (Cat. 9271 Cell Signaling Technology, Danvers, MA, USA), Akt (pan) (C67E7; Cat. 4691, Cell Signaling Technology), Fatty Acid Synthase (G-11; Cat. sc-48357, Santa Cruz Biotechnology, Dallas, TX, USA), C/EBPα (14AA; Cat. sc-61, Santa Cruz Biotechnology), anti-FABP4 (Cat. ab195657, Abcam, Waltham, MA, USA), and Perilipin-1 (N-terminus; Cat. GP29, Progen Biotechnik, Heidelberg, Germany). Secondary antibodies coupled to IRDye-680 (Cat. 926-68023, LI-COR Biosciences, Lincoln, NE, USA), IRDye-800 (Cat. 926-32214, LI-COR Biosciences), or horseradish peroxidase (Cat. 111-035-144 and 115-035-146, Jackson ImmunoResearch, West Grove, PA, USA) were incubated for 1 h at room temperature, and blots were visualized using Bio-Rad ChemiDoc MP Imaging System (Bio-Rad Laboratories). Fiji ImageJ2 software was used for densitometric analysis of protein bands [43].

### 2.4. Insulin Sensitivity Assay

Cells were treated as described in each experiment, and 18 h before harvest, medium was replaced with DMEM/F12 plus 0.2% BSA (Cat. A9647, Sigma-Aldrich) to serum-deprive before performing a 10-min treatment with 10 nM insulin (Cat. I9278, Sigma-Aldrich). Cells were then harvested for protein quantification and Western blot analysis of phospho-Akt Ser473 and Akt (pan) levels (antibodies indicated above).

### 2.5. Lipid Droplet Staining and Microscopy

For Oil Red O staining, fixation and staining were performed directly on the wells of cell culture plates. Cells were washed 3 times with PBS and fixed for 20 min with 4% paraformaldehyde (PFA) and 4% sucrose in PBS. Fixed cells were washed twice with PBS and stained with 2 mg/mL Oil Red O solution for 30 min. Excess dye was removed by washing with water, and images were taken using a hand-held camera. Alternatively, indirect immunofluorescence was performed as previously described [44]. After fixation, permeabilization, and blocking, cells were incubated with 1 µg/mL BODIPY 493/503 (Cat. 25892, Cayman Chemical) in PBS 0.1% Tween20 2% BSA for 30 min at room temperature. After three washes with the same buffer, cells were mounted on glass slides with VECTASHIELD Antifade Mounting Medium with DAPI (Cat. H-1200-10, Vector Laboratories, Newark, CA, USA). Images were taken on a Zeiss LSM 710 laser scanning confocal microscope (Carl Zeiss, Oberkochen, Germany), and LD size was assessed with Fiji ImageJ2 software.

### 2.6. RNA-Sequencing

mRNA was prepared for sequencing from total RNA samples using TruSeq Stranded mRNA library kit from Illumina (San Diego, CA, USA). Sequencing was performed using the NovaSeq platform with 50 bp paired-end reads at the Norwegian Sequencing Center. RNA-seq reads were filtered to remove low-quality reads by Trimmomatic v0.39 [45]. Filtered reads were aligned to hg38 genome with STAR v2.7.10a using Ensembl GRCh38.p13 release 104 reference annotation [46]. Transcript abundance was estimated using featureCounts in Subread v2.0.1 [47]. DESeq2 (v 1.36.0) [48], as implemented in SARTools [49], was used to normalize the raw counts, apply exploratory analysis (e.g., principal component analysis), and to perform differential expression analysis. Reads per kilobase of transcript per million mapped reads (RPKM) were calculated for each transcript.

### 2.7. GSEA with the MSigDB Hallmark Collection

Gene set enrichment analysis (GSEA) was performed on normalized read counts [50]. Gene ranking was generated across each comparison with Pearson correlation metric and analyzed against the mSigDB Hallmark v7.5.1 gene sets [51].

### 2.8. Statistical Analyses

All statistical analyses except for the RNA-sequencing data were performed in GraphPad Prism v. 10.2.0. The statistical tests and *p* values are listed in the figure legends. Before statistical testing, data representing different biological replicates were normalized to each other using the Solver Add-in in Microsoft Excel, with the *Set Objective*: Minimum Sum of Relative Standard Deviation (RSD) and *Solver Method* (iterations): Generalized Reduced Gradient (GRG) Nonlinear.

### 2.9. Data Availability

RNA-seq data generated for this study are available at NCBI GEO with accession number GSE280110.

## 3. Results

### 3.1. Repeated Cycles of In Vitro Fasting and Refeeding Elicit Changes in the Expression of Adipogenic Genes

**Figure 1:**
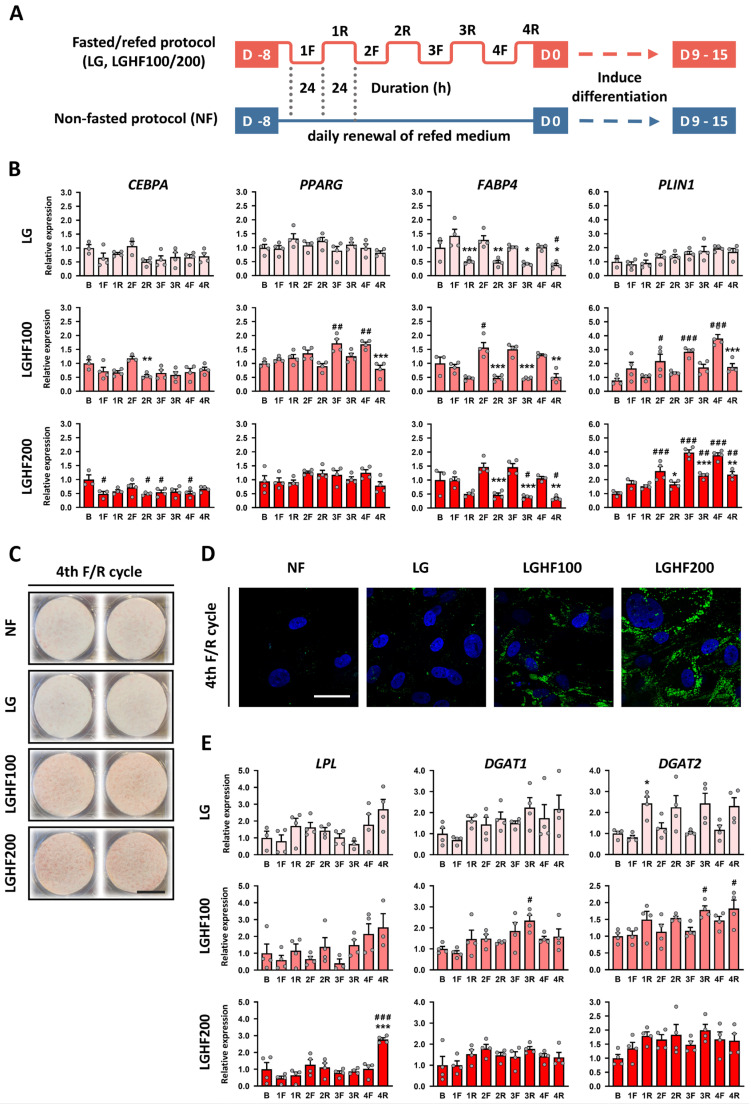
Repeated cycles of in vitro fasting and refeeding elicit changes in the expression of adipogenic genes and increase the storage of neutral lipids in ASCs. (**A**) Experimental design: Human adipose stem cells (ASCs) were fasted for 24 h in low glucose (LG; 5 mM glucose) or low glucose/high fatty acid medium (LGHF100/200; 5 mM glucose + 100 or 200 μM oleic acid) and then refed for 24 h with high glucose/low fatty acid medium (HGLF; 17.5 mM glucose). This was performed four times. For the non-fasted protocol (NF), the cells were cultured in HGLF medium for 8 days, with daily medium changes. (**B**) Relative expression of adipogenic marker genes in non-proliferating ASCs being subjected to different fasting and refeeding regimens. ASCs that had gone through four fasting and refeeding cycles were fixed with paraformaldehyde and neutral lipids stained with (**C**) Oil Red O, scale bar: 1 cm, or (**D**) BODIPY, scale bar: 40 µM. The pictures shown are representative of three biological replicates. (**E**) Relative expression of esterification genes. The gene expression data are presented as mean fold-change ± SEM of 3–4 biological replicates. Fasted (F) vs. Refed (R): * *p* < 0.05, ** *p* < 0.01, *** *p* < 0.001 and Basal (B) vs. F or R: ^#^ *p* < 0.05, ^##^ *p* < 0.01, ^###^ *p* < 0.001, determined by one-way ANOVA with Tukey’s multiple comparison test. NF: non-fasted; LG: low glucose; LGHF100/200: low glucose/high fatty acid (100/200 μM oleic acid).

To address potential phenotypic and transcriptional effects of EODF, we examined how human ASCs responded to a regimen of repeated exposure to fasting media with low glucose (LG) concentration and high FA (HF) concentrations (LGHF), followed by high glucose/low FA (HGLF) inherent in regular/standard growth media. To this end, cells were cultivated to confluency in HGLF (17.5 mM glucose; 10% fetal bovine serum [FBS]) and then changed to fasting medium (F; Figure 1A; 5 mM glucose; 2% FBS) with or without 100 or 200 μM oleic acid (LG or LGHF100/200) to simulate the effect of fasting lipolysis. After 24 h of incubation in fasting conditions, cells were changed back to HGLF refeeding medium for 24 h. This cycle was repeated three additional times (Figure 1A).

This alternating “fasting/refeeding” (F/R) treatment elicited cyclic expression of the adipogenic marker genes *FABP4*, an intracellular lipid-binding and transport protein, and *PLIN1*, a lipid droplet-binding regulator of lipolysis. *FABP4* and *PLIN1* tended to be induced, or were significantly upregulated, under fasting conditions, especially with LGHF100/200 (Figure 1B).

Interestingly, this nutrient cycling seems to produce opposing changes in expression levels under refed conditions (Figure 1B), with *FABP4* being downregulated and *PLIN1* upregulated, relative to baseline (B) by the end of the four F/R cycles. *PPARG2* and *CEBPA* were less affected by F/R cycling, except for *PPARG2* when using LGHF100. In this case, the observed behavior was that of *FABP4* and *PLIN1*.

### 3.2. Increased Storage of Neutral Lipids After Repeated F/R Cycles

While mature adipocytes rely on lipolysis and re-esterification of TAGs as substrate for lipid storage, progenitors have a limited ability to esterify or re-esterify FAs into TAG [52,53]. Still, ASCs that have gone through four F/R cycles with LGHF100/200 showed increased Oil Red O staining (Figure 1C), which was substantiated by BODIPY staining (Figure 1D). Curiously, this seems to be paralleled by increased expression of genes involved in esterification, like *LPL*, *DGAT1*, and *DGAT2* (Figure 1E). Taken together, these findings indicate that repeated F/R cycles might enable ASCs to esterify some of the exogenous FAs, in our case oleic acid (OA), into TAGs. This cellular response is, in fact, more similar to what is observed with differentiating or fully differentiated adipocytes that are challenged with high concentrations of OA to model adipocyte hypertrophy or to evaluate the effects of FA saturation on lipid droplet dynamics [54,55].

### 3.3. ASCs Exposed to F/R Cycles Display a Lipolytic/Re-Esterifying Gene Expression Profile During Adipogenic Differentiation

**Figure 2:**
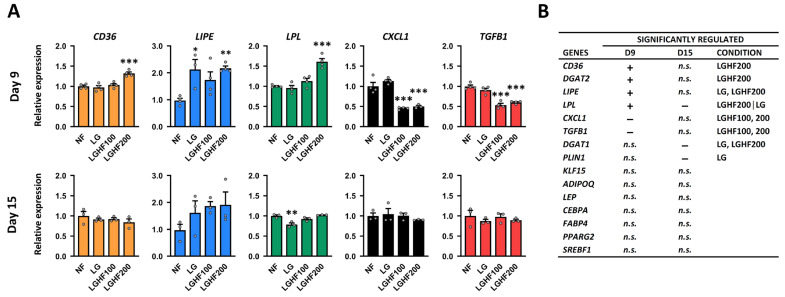
F/R cycled ASCs display a lipolytic/re-esterifying gene profile during adipogenic differentiation. (**A**) Expression of selected genes in differentiating ASCs subjected to prior fasting and refeeding regimens relative to NF cells. (**B**) Table view of all genes analyzed by qPCR being significantly upregulated (+), downregulated (−) or unaffected (n.s.) in F/R cycled adipocytes on day 9 or day 15 of differentiation relative to NF cells. The corresponding graphs can be found in Figure S1. The data are presented as mean fold-change ± SEM of 3–4 biological replicates. * *p* < 0.05, ** *p* < 0.01, *** *p* < 0.001 as determined by one-way ANOVA with Dunnett’s multiple comparison test. NF: non-fasted; LG: low glucose; LGHF100/200: low glucose/high fatty acid (100/200 μM oleic acid); n.s: non-significant.

To assess whether repeated F/R cycles affected the ASC adipogenic potential, we differentiated ASCs subjected to four F/R-cycles (8 days total) with or without OA and compared these with “non-cycled” ASCs cultured in standard medium (HGLF) for 8 days prior to differentiation (NF, non-fasted). Cells were examined on day 9 and day 15 of differentiation. Quantitative PCR showed that the cells exposed to OA during the F/R cycles (LGHF200) had a significant increase in the expression of *LPL*, *LIPE*, and *CD36* on day 9 (Figure 2A)*,* all involved in FFA pathways in the cell and known to be upregulated by fasting and low insulin [56]. At the same time, LGHF cells showed a lower expression of *CXCL1* and *TGFB1* compared to NF and LG cells (Figure 2A). These represented about one-third of all genes assessed by RT-qPCR (Supplementary Figure S1). Of note, effects on gene expression seen on day 9 were lost by day 15 (Figure 2A,B). Together, these results suggest that cyclic changes in nutrient availability associated with intermittent fasting can affect gene expression in differentiating adipocytes in the medium term. However, these changes are reversed in the long term as the cells progress to a more mature adipogenic state.

### 3.4. Lipid Droplet Dynamics During Differentiation of Fasted and Refed ASCs

**Figure 3:**
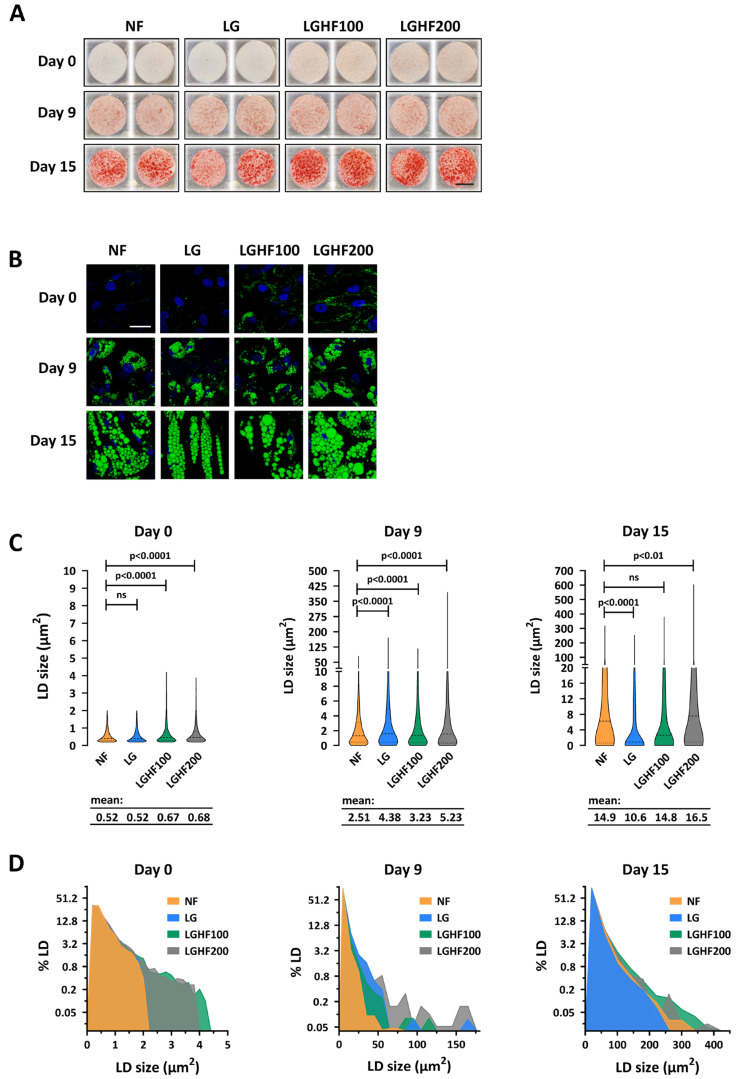
Lipid droplet dynamics during differentiation of fasted and refed ASCs. Differentiating ASCs subjected to prior fasting and refeeding regimens were fixed with paraformaldehyde on day 0, 9,and 15 after start of differentiation, and neutral lipids stained with (**A**) Oil Red O, scale bar: 1 cm, or (**B**) BODIPY, scale bar: 40 µM. Lipid droplets (LDs) were quantified using Fiji and presented as (**C**) violin plots (median + quartiles) and (**D**) frequency plots (bin width day 0: 0.2 μm^2^; day 9: 10 μm^2^; day 15: 40 μm^2^). The plots are calculated based on three biological replicates. The *Y*-axes are plotted with log2 scale, and the Day 9 *X*-axis cropped at 180 μm^2^ to highlight the differences. Significance levels were determined by unpaired, two-tailed *t* test. NF: non-fasted; LG: low glucose; LGHF100/200: low glucose/high fatty acid (100/200 μM oleic acid).

Since we observed a difference in intracellular neutral lipid content in ASCs after four F/R cycles (see Figure 1C,D and Figure 3C,D), we asked whether this difference would persist or increase during differentiation. We observed different lipid droplet (LD) dynamics depending on whether the cells had been fasted with LG or LGHF100/200 (Figure 3A,B). While the F/R cells on average had a larger mean size of LDs than the non-cycled cells on day 9 of differentiation (Figure 3C; 3.23–5.23 vs. 2.51 μm^2^), only the LGHF200 cells still showed augmented LD size on day 15 (16.5 vs. 14.9 μm^2^) (Figure 3C). The LDs in the LGHF100 cells were now comparable with non-cycled cells, while the LG cells displayed LDs that on average were smaller than in the NF group (10.6 vs. 14.9 μm^2^; Figure 3C). The increase in the average LD size for the LGHF200-cycled cells on day 15 indicated that these cells were affected, at least metabolically, by events more than 2 weeks earlier, despite gene expression levels being back to normal (see Figure 2A). Notably, only a minor fraction (Day 0: ~9.5%; Day 9: ~8%; Day 15: ~1.5%) of the LDs were larger in LGHF200 cells when compared to NF cells by calculating ΔAUC (Figure 3D). Still, this indicates that a subset of the cells and/or LDs had reached a threshold that triggered semi-permanent changes.

### 3.5. Insulin Sensitivity Is Reduced in Adipocytes During Differentiation, Independently of Prior Fasting and Refeeding

**Figure 4:**
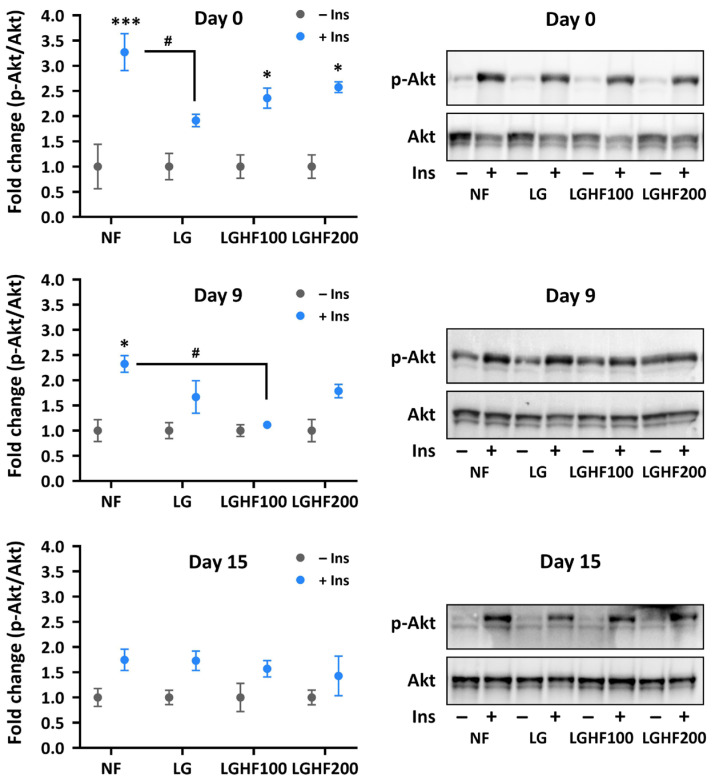
Insulin sensitivity is reduced in adipocytes during adipogenic differentiation, independent of prior fasting and refeeding. Differentiating ASCs subjected or not to prior fasting and refeeding regimens were serum-deprived overnight and stimulated with 10 nM insulin for 10 min on day 0, 9, and 15 after start of differentiation. The relative level of phosphorylated Akt was determined by immunoblotting with phoshpo-Ser473 and total Akt antibodies and densitometric quantification of the resulting blots. The data are presented as mean p-Akt/Akt ratio ± SEM of 2–3 biological replicates. One representative immunoblot is shown per time point. Insulin-stimulated vs. non-stimulated: * *p* < 0.05, *** *p* < 0.001, and insulin-stimulated vs. insulin-stimulated between different F/R regimens: ^#^ *p* < 0.05, as determined by two-way ANOVA with Tukey’s multiple comparisons test. Only significant differences are indicated in the figure. NF: non-fasted; LG: low glucose; LGHF100/200: low glucose/high fatty acid (100/200 μM oleic acid).

Adipocyte size and lipid droplet morphology correlate with systemic insulin sensitivity [57,58] and adipocytes’ ability to respond adequately to insulin [59,60]. Using the phospho-Ser473 Akt to total Akt ratio (p-Akt/Akt) as a proxy for insulin sensitivity, we found the non-cycled ASCs to be the most insulin-sensitive on day 0, followed by LGHF100/200 and LG-fasted cells (Figure 4). On day 9, and even more so on day 15, the differences between the F/R regimens were reduced (Figure 4).

### 3.6. F/R Cycling-Dependent Activation of Functional Pathways Is Lost by Day 15 of Differentiation

**Figure 5:**
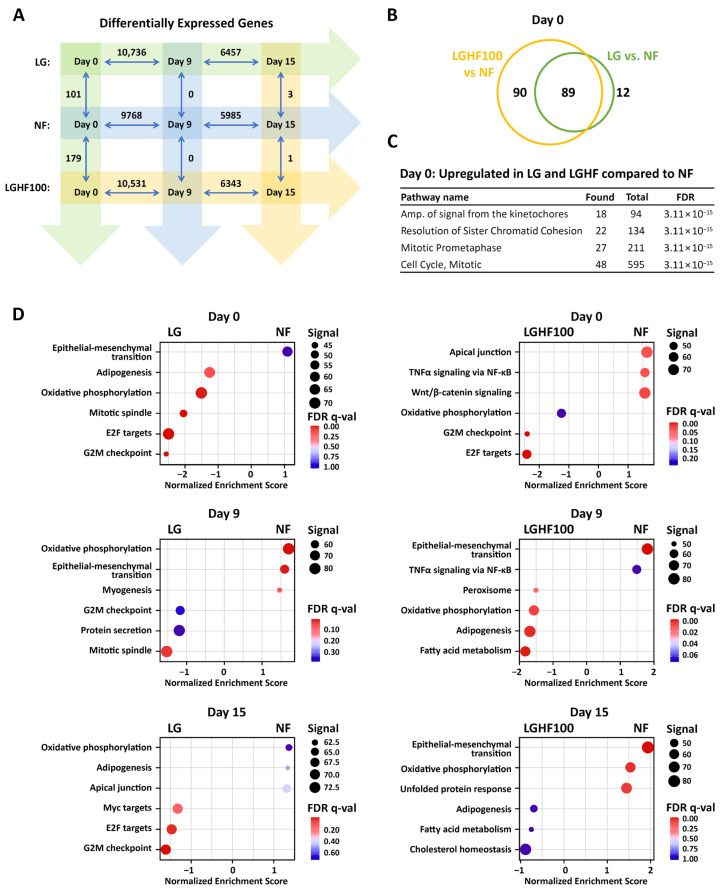
F/R cycling-dependent activation of functional pathways are lost by day 15 of differentiation. Total RNA sequencing was performed on ASCs at baseline (Day 0; *n* = 5, 3 donors), following either four F/R cycles or 8 days in standard culture medium, and after consecutive induction of differentiation on Day 9 and Day 15 under the same prior conditions (*n* = 3, 1 donor). (**A**) Differentially expressed genes (DEGs) between time points and pre-treatments in differentiating ASCs (FDR-adjusted *p*-value < 0.05). (**B**) Venn diagram of DEGs in LG- and LGHF100-cycled compared to NF cells on day 0. (**C**) The intersecting DEGs from (B) were tested for overrepresentation against the Reactome gene sets. (**D**) Gene set enrichment analysis (GSEA) with the MSigDB Hallmark gene set. NF: non-fasted; LG: low glucose; LGHF100: low glucose/high fatty acid (100 μM oleic acid).

To obtain a transcriptome-wide insight into the pathways elicited by the various F/R regimes, we sequenced total RNA from adipocytes on day 0 (after four F/R cycles) and on day 9 and 15 of differentiation. As control, we used RNA from non-F/R-cycled cells, at the same time points. As expected, we found thousands of differentially expressed genes (DEGs) during differentiation, regardless of F/R regimen (Figure 5A; D9 and D15 vs. D0). Strikingly however, we identified relatively few DEGs between treatments, and primarily at the undifferentiated stage (D0), where 101 and 179 DEGs were identified when comparing non-cycled cells with LG- and LGHF100-cycled cells, respectively (Figure 5A; Supplementary Table S2). Most of these genes were upregulated by prior F/R cycles (LG-cycled: 94 genes; LGHF100-cycled: 157 genes). Moreover, 89 of these genes were common between the LG and LGHF100 cells. (Figure 5B). Gene ontology (GO) enrichment analyses of the union DEGs on day 0 revealed involvement in mitotic processes (Figure 5C). Among the five upregulated genes in LGHF100-cycled cells, compared to LG-cycled cells, we found *PDK4* and *PLIN2* (Supplementary Table S2), which have been shown to be induced during lipolysis and by lipolytic agents in brown adipocytes [61].

Gene set enrichment analysis (GSEA) showed enrichment of such hallmarks as *Mitotic spindle*, *G2M checkpoint* and *E2F targets* for the F/R-cycled cells in LG and LG100-cycled cells on day 0 (Figure 5D), supporting the GO enrichment analysis (Figure 5C). In our analysis of the DEGs, we effectively found none between conditions after differentiation onset (Figure 5A; days 9 and 15). Nevertheless, both the *Adipogenesis* and *Oxidative phosphorylation* hallmarks were enriched in the LG-cycled compared to NF cells on day 0 (Figure 5D), while the corresponding overrepresentation in LGHF100-cycled cells was seen on day 9. On day 15, enrichment of *Adipogenesis* genes between all was lost in both LG and LGHF100-cycled cells, while *Oxidative phosphorylation* genes were overrepresented in the transcriptome of NF cells when compared to LGHF100-cycled cells (Figure 5D).

We conclude that whereas F/R regimes elicit most gene expression changes in undifferentiated cells (day 0), the numbers of DEGs are relatively low and decrease over time during differentiation. Our RNA-seq data support and extend our initial RT-qPCR results and suggest that F/R-elicited transcriptional changes in ASCs fade once they start to differentiate.

## 4. Discussion

Changing the quantity and timing of nutrient intake by intermittent fasting has multiple beneficial health effects [2,3,4]. However, how recurrent fasting affects the differentiation and lipid-storing capacity of ASCs is less known. Here, we show that, by mimicking the biochemical changes in the adipose niche in vitro during repeated fasting and refeeding, we were able to impose transcriptional changes for essential adipogenic markers. Furthermore, lipid storage in the form of LDs increased and was accompanied by upregulation of lipogenic genes for the LG-cycled cells and/or FA esterification genes for the LGHF-cycled cells. Expression levels of genes involved in the regulation of cell cycle progression were also gradually altered after four consecutive F/R rounds compared to non-fasted ASCs. Importantly, differentiation of ASCs to mature adipocytes overrides or erases the changes imposed by the F/R cycles, and most of the differences between cycled and non-cycled cells are lost. This highlights the robustness of adipocytes, in line with current ideas about cellular resilience [62,63] and adipogenic memory [64].

Studying the effects of fasting and refeeding in the adipose niche is mainly performed in vivo in mice. However, the desire to dissect the contribution from metabolites, hormones, and neurotransmitters on the different cell types in human adipose tissue has encouraged the use of alternative strategies. As an integrated adipose response, lipolysis can be induced neurologically by catecholamines [65,66]; endocrinologically by lowering the insulin:glucagon ratio [67]; nutritionally by fasting, i.e., reducing insulin [68]; or chemically by, e.g., ABHD5 ligands (SR-3420/SR-4995) [61,69]. All these approaches are dependent on available lipid reservoirs, i.e., mature, lipid-filled adipocytes, and do not permit studies of the metabolic effects of lipolysis exclusively on ASCs. Hence, in our system, we chose to mimic the local effects of repeated bouts of fasting and refeeding by stimulating ASCs with low serum, low glucose, and high levels of FAs. The induction of the lipolytic marker genes *PLIN2* and *PDK4* in the LGHF-cycled cells (Supplementary Table S2) underscores the difference between fasting ASCs with or without fatty acid (LG vs. LGHF) in the fasting media, and provides additional evidence for the relevance of our model in mimicking features of fasting-induced metabolic changes in ASCs [61].

Following four F/R cycles, we observed an activation of adipogenic genes and genes involved in FA esterification, in parallel with an increased storage of neutral lipids (Figure 1). The effect was most pronounced in cells fasted with LGHF medium. This suggests that these cells, through currently unknown mechanisms, are more predisposed to store energy, either by lipogenesis or by esterification. FAs, especially unsaturated FAs and hydroxyl-FAs, are known to activate PPARγ [39,40,41], which in turn induces genes involved in both de novo lipogenesis and (re-)esterification [70,71]. Therefore, the increased LD size observed in the LGHF-cycled cells on days 0 and 9 of differentiation (Figure 1 and Figure 3) could be attributed to one or both metabolic pathways. Our assays did not allow us to discriminate between these two alternatives, and tracing studies with radiolabeled OAs would be the next step to disentangle these two pathways [72,73].

Concurrent with the increased incorporation of neutral lipids in the LGHF-cycled ASCs, we observed an upregulation of pathways involved in mitotic checkpoints such as *Amplification of signal from the kinetochores*, *Mitotic spindle checkpoint*, and *Separation of sister chromatids* (Figure 5). Before F/R cycling, ASCs were cultured to confluency in the presence of basic fibroblast growth factor (FGF2; Figure 1A). Upon initiation of F/R cycling, FGF2 was removed, keeping cells in a non-proliferative state due to contact inhibition [74]. This differs from conditions in adipose tissue, where ASCs primed for differentiation retain the ability to proliferate. Interestingly, intermittent fasting is known to increase proliferation in other metabolically active organs, such as liver, brain, and intestine [75,76,77].

Adipose tissue and its inherent processes, such as lipogenesis, esterification, lipolysis, and re-esterification, have evolved as an adaptation to variable energy availability [78]. Healthy adipose tissue responds to fasting by increasing lipolysis to mobilize FAs for hepatic β-oxidation [31,32]. During the EODF regimen, the mature adipocyte would rhythmically alternate between storing and mobilizing FAs. In mice, EODF has been shown to elicit browning of visceral WAT [12]. This was suggested to result from remodeling of gut microbiota and increased lactate and acetate in circulation, although this link was not formally proven. Lactate and acetate have been suggested to induce browning through UCP-1 expression [12,79]. Could an increase in FFA locally in the WAT contribute to preparing progenitors for browning during EODF, via, e.g., activation of PPARγ? The primary cells used in the current study are able to beige using rosiglitazone, a PPARγ agonist [80] but did not show signs of browning/beiging by induction of brown/beige markers, e.g., *UCP1*, *CIDEA*, *PPARGC1A*, *PPARG*, *CITED1*, and *SLC27A1* [81,82], upon in vitro EODF (Supplementary Table S2). Two exceptions to this are *PDK4* and *PLIN2*, which showed an increase in LGHF100 relative to LG-cycled cells on day 0. Both genes play an important role in brown adipocytes and are overexpressed in brown as compared to white adipocytes and induced during cold stimulation [83,84]. As brown adipocytes have been shown to improve insulin-stimulated glucose uptake in vivo into both brown and white adipose tissue [85], it is important to note that there seem to be no beneficial effects of the EODF regimens on insulin sensitivity in vitro (Figure 4).

Our data support a view of different types of memories: transcriptional memory [86], epigenetic memory [87], adipogenic memory [64,88,89], and metabolic memory [90,91]. During recurrent F/R bouts, expression of several genes, including *FABP4*, *PLIN1*, and *LPL*, tended to increase or decrease progressively from cycle to cycle, suggesting that elements of transcriptional and/or epigenetic memory might be at play [92]. Moreover, the average size of lipid droplets in maturing adipocytes was significantly larger on day 9 for the F/R-cycled cells and to a certain degree on day 15 for the LGHF200-cycled cells. This suggests that cells may have retained an adipogenic or metabolic memory of the previous F/R regime, even though transcriptional memory is lost. These observations may be explained by the long half-life of proteins in fatty acid storage and metabolism pathways, reducing the need for sustained transcription. On day 15, most if not all, transcriptional and metabolic memory of the EODF regimen seemed to have been erased.

At the intersection between adaptation (memory) and recovery (memory erasure), cells must determine whether to return to a homeostatic state (stem cells) or to transition into a differentiation trajectory [63]. In this context, we note that gene programs such as oxidative phosphorylation, which is crucial for adipocyte differentiation [93,94], alternated between being enriched and underrepresented in LG and LGHF100 cells during progression from day 0 to day 9 and day 15. This suggests a resilience behavior where robust, opposing processes or pathways are activated to restore homeostasis [62]—in our case, a non-proliferative, adipogenic, lipid-storing state—after a perturbation such as intermittent fasting. Within the boundaries defined by the in vitro fasting and refeeding regimen described in this article, this appears to be the preferred outcome.

## 5. Conclusions

This study reveals the transient effects of every-other-day fasting (EODF) on human adipose stem cells (ASCs). Repeated fasting and refeeding cycles significantly impact adipogenic gene expression and lipid storage. However, these fasting-induced changes are not sustained over time, as ASCs display resilience, returning to their physiological trajectory during differentiation. These findings indicate that while EODF initially modulates ASC behavior and metabolism, its long-term effects on adipose tissue homeostasis may be limited. This underscores the adaptability of adipose tissue and provides valuable insights into the therapeutic potential of intermittent fasting strategies.

## Data Availability

RNA-seq data generated for this study is available at NCBI GEO with accession number GSE280110.

## Supplementary Materials

The following supporting information can be downloaded at: https://www.mdpi.com/article/10.3390/nu16244310/s1, Figure S1: Relative expression of adipogenic and lipolytic genes during differentiation; Figure S2: Immunoblot analysis of adipogenic factors; Table S1: Primer list; Table S2: Differentially expressed gene lists.
