# Supplementary material for: Transcriptional and Metabolic Changes Following Repeated Fasting and Refeeding of Adipose Stem Cells Highlight Adipose Tissue Resilience
Source: Nutrients. 2024 Dec 13;16(24):4310. doi: 10.3390/nu16244310 (PMC11676188; doi:10.3390/nu16244310)
Supplement: Supplementary file 1 [file nutrients-16-04310-s001.zip › Figure S1.pdf]

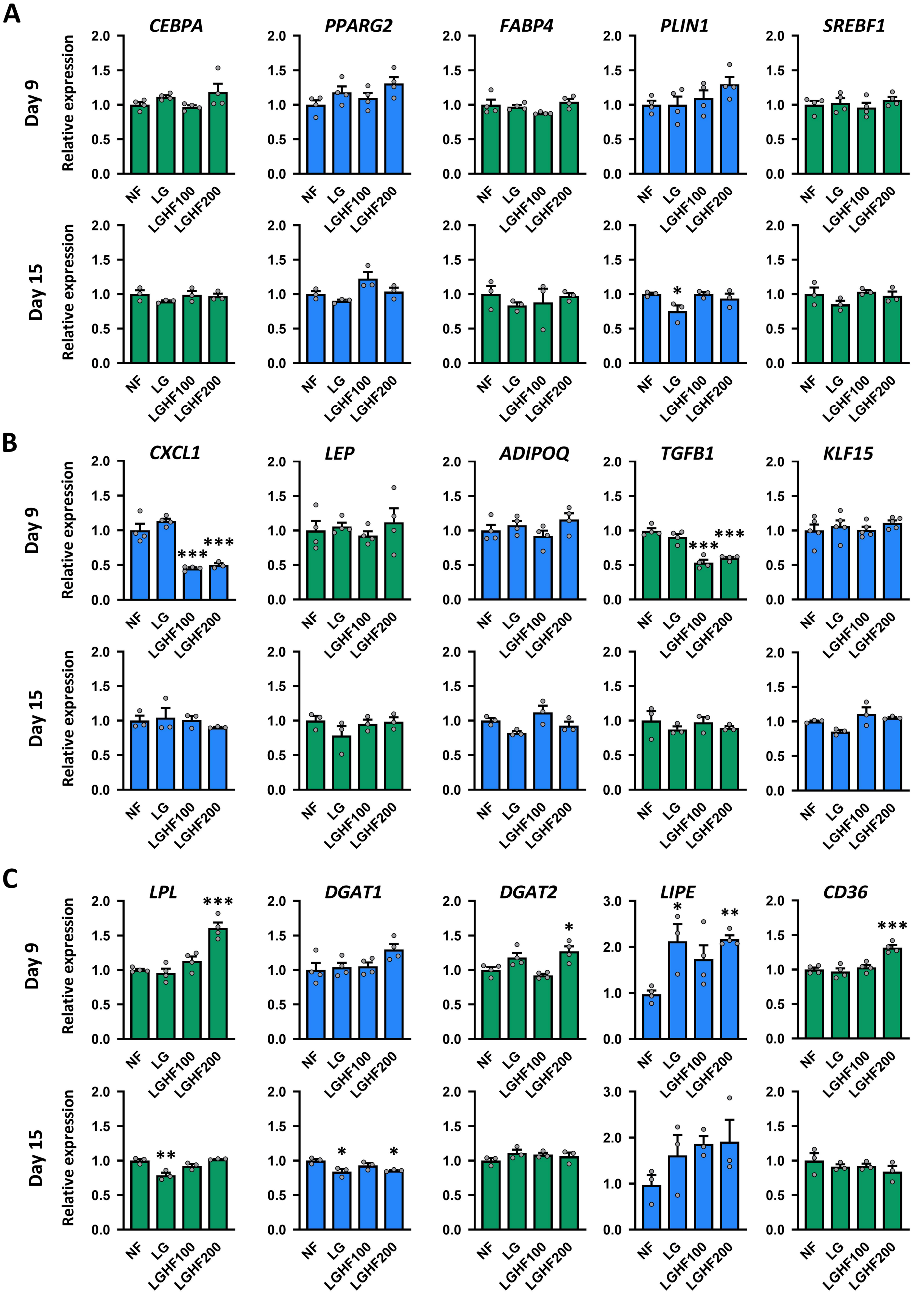

Figure S1. Fasted and refed ASCs display a lipolytic gene profile during adipogenic differentiation. Relative expression of (**A**) adipogenesis, (**B**) cytokine/endocrine, and (**C**) TAG and lipid metabolism genes in differentiating ASCs subjected or not to prior fasting and refeeding regimens. The data are presented as mean fold-difference ± SEM of 3-4 biological replicates. *$p < 0.05$, **$p < 0.01$, ***$p < 0.001$, as determined by one-way ANOVA with Dunnett's multiple comparison test. NF: non-fasted; LG: low glucose; LGHF100/200: low glucose/high fatty acid (100/200 μM oleic acid).
