# Supplementary material for: Transcriptional and Metabolic Changes Following Repeated Fasting and Refeeding of Adipose Stem Cells Highlight Adipose Tissue Resilience
Source: Nutrients. 2024 Dec 13;16(24):4310. doi: 10.3390/nu16244310 (PMC11676188; doi:10.3390/nu16244310)
Supplement: Supplementary file 1 [file nutrients-16-04310-s001.zip › Figure S2.pdf]

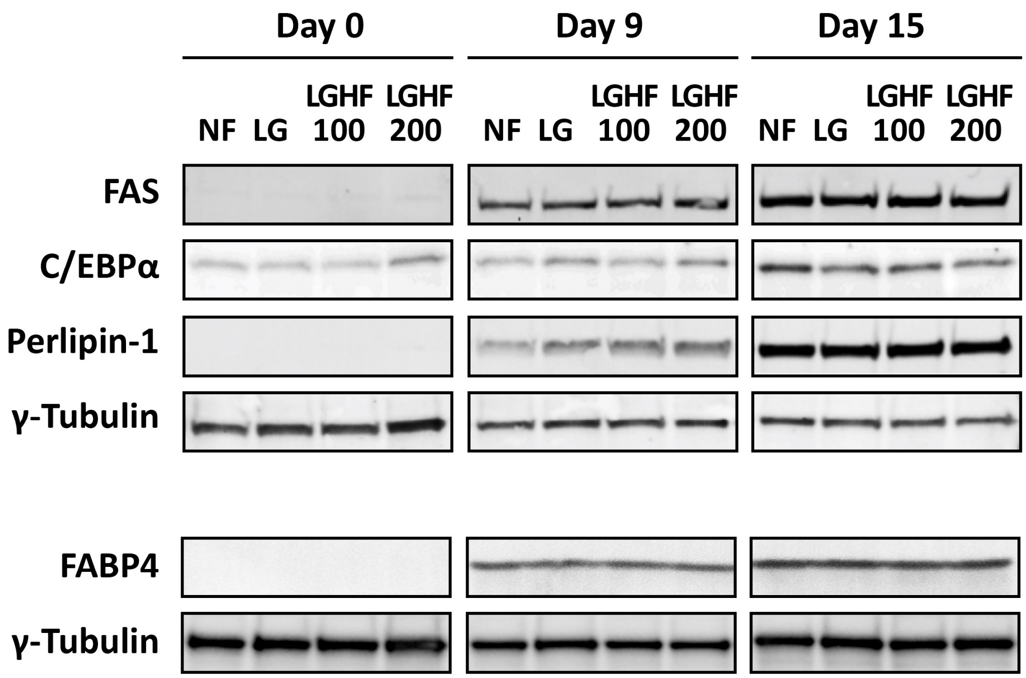

Figure S2. Immunoblot analysis of adipogenic factors in differentiating ASCs on day 0, 9 and 15 after start of differentiation, subjected or not to prior fasting and refeeding regimens. γ-Tubulin was used as internal reference. The presented blots are representative for three independent immunoblots. NF: non-fasted; LG: low glucose; LGHF100/200: low glucose/high fatty acid (100/200 μM oleic acid).
